# Sociodemographic Characteristics of Tobacco Consumers in a Rural Area of Bangladesh

**Published:** 2007-12

**Authors:** Kaneta Choudhury, S.M.A. Hanifi, Shehrin Shaila Mahmood, Abbas Bhuiya

**Affiliations:** Social and Behavioural Sciences Unit, Public Health Sciences Division, ICDDR,B, GPO Box 128, Dhaka 1000, Bangladesh

**Keywords:** Health outcomes, Tobacco, Tobacco consumption, Bangladesh

## Abstract

Bangladesh typifies many developing countries experiencing an increasing trend in tobacco consumption. However, little is known about the general pattern of tobacco consumption and about population groups who are more prone to tobacco consumption. This paper aimed at generating knowledge on tobacco consumption, especially emphasizing the identification of sociodemographic groups who are more prone to tobacco consumption vis-à-vis tobacco-related health consequences in a remote rural area in Bangladesh. Information on the tobacco consumption status of 6,618 individuals (52.1% males, 47.9% females), aged over 15 years, was collected in 1994. Both univariate and multivariate analyses were done. Individuals were categorized as consumers if they consumed tobacco in any form at all, i.e. smoke or chew. The independent variables included various characteristics of individuals and households. Overall, 43.4% of the study subjects consumed tobacco. Males were 9.38 times more likely to consume tobacco than their female counterparts. Individuals with no education were 3.62 times more likely to consume tobacco than those who had completed six or more years of schooling, and the poor were almost twice as likely to consume tobacco than the rich. Tobacco consumption in both smoke and chewing form has been a part of household consumption in Bangladesh from time immemorial. Only aggressive anti-tobacco programmes on various fronts may salvage the vulnerable groups from the menace of tobacco consumption in Bangladesh.

## INTRODUCTION

Tobacco-use results in both health and economic costs that are large and growing. Smoking kills one in 10 people worldwide; one in two long-term smokers dies from the habit (1,2). By 2030, smoking is expected to be the single biggest cause of death worldwide, resulting in one in six deaths and killing 10 million people (3). Apart from premature mortality, prolonged smoking also causes considerable disability (3).

Results of research indicate that cigarette smoking causes cancer of the lung, larynx, oral cavity, and oesophagus and is significantly associated with pancreas, urinary bladder, stomach, and kidney cancers in both men and women (4,3). Smoking is also associated with ischaemic heart disease and other vascular diseases, including heart attacks, strokes, and other diseases of the arteries or veins and is the leading cause of chronic bronchitis and emphysema (3–7).

About one-third of the global population aged over 15 years—1.1 billion people—are smokers. Of these smokers, 800 million are in developing countries (3). Existing data suggest that, globally, men are more likely to smoke than women (47% vs 12%) (4). Men from the developing world smoke more in proportion (48%) than men from the developed world (42%). However, the pattern is opposite for women—7% in the developing world versus 24% in the developed world. Although the impact of tobacco-related diseases and death had until recently been a problem primarily for developed countries, smoking has, to some extent, been brought under control through tougher legislation and increasing awareness in the developed world (4).

Transnational tobacco companies are now aggressively targeting developing countries, including Bangladesh (3), where health information is less well-known, to promote smoking (8). The World Health Organization estimates that, by the mid-2020s, about 85% of the world's smokers will be in the poorer countries, and seven in every 10 tobacco-related deaths will be in these countries (3).

Bangladesh is already experiencing an increasing number of cancer cases; there was a 10-fold increase of cases between 1960 and 1980. Part of the reason for this increase could also be the availability of better diagnosis and/or increased contact with healthcare providers. However, the Bangladesh Cancer Society estimates that a significant proportion of all cancers in Bangladesh are related to tobacco (9,10). A study in Dhaka concluded that tobacco consumption—either through chewing or smoking—was an important factor in the development of oral cancer (11). Smoking has also been named as an important risk factor for heart disease among male patients in their 40s and 50s in Bangladesh (9).

Information on the general pattern of tobacco consumption in Bangladesh is sparse. Little is known about the vulnerability of any sociodemographic group to tobacco consumption and related health consequences. If a particular group of population is more inclined towards tobacco consumption, that group will suffer most from the effects leading to an inequitable situation. Thus, it is important to know the existence of any vulnerable groups to develop appropriate targeted interventions to reduce inequity in the society.

This paper aimed at generating knowledge on tobacco consumption with special emphasis on the identification of sociodemographic groups who are more prone to tobacco consumption vis-à-vis tobacco-related health consequences in a remote rural area in Bangladesh.

## MATERIALS AND METHODS

### Study area

The study was carried out in Chakaria upazila of Cox's Bazar district, which is located in the southeast coast of the Bay of Bengal. The area was typically characterized as one of the most traditional in terms of religion and openness to new ideas (12–15). Nearly half of males and two-thirds of females aged over six years had never been to school. It was also recognized as one of the most impoverished in terms of health, family planning, and NGO activities (14). Cultivation of tobacco is a common sight in parts of Chakaria.

### Data collection

Data were collected from Chakaria upazila of Cox's Bazar district during October-December 1994 as part of the baseline survey of the Chakaria Community Health Project of ICDDR,B. The survey included all the first-phase unions (the lowest administrative unit of the Government), namely Baraitali, Kayerbeel, BM Char, Harbang, and Purbo Bheola, of the Project. The unions were purposively selected from unions that were far from, not-so-far from, and close to the Chakaria headquarters. During the study year, the five unions had a population of around 120,000. The survey covered all the villages in the unions.

A systematic random sample of 12% of 17,608 households was selected for the survey. In the case of absentees or refusals, the adjacent comparable household made replacement.

The questionnaire was administered to the head of the household or any other senior person of the household to collect information on individuals and on household characteristics. Information on tobacco consumption was collected for 3,448 men and 3,170 women aged over 15 years.

### Variables

The survey had asked whether individuals smoked or consumed tobacco; this yielded ‘yes’ or ‘no’ response. If the response was ‘yes‘, details of what they smoked or chewed were asked, i.e. cigarettes, *bidis*, and *hookah*, or *shada* (dried tobacco leaf) or *zarda* (processed tobacco leaf in a paste), which are both chewed. *Bidis* consist of sun-dried and cured tobacco flakes hand-rolled in a rectangular piece of paper or tobacco leaf; these are generally very inexpensive costing only a fraction of the price of cigarettes and deliver large amounts of tar and nicotine. Cigarettes of local producers generally also contain high tar and nicotine. With the *hookah*, which is rare, tobacco mixed with molasses is burnt, and the smoke passed through water in a specially-devised tool before inhalation. The smokeless forms of tobacco commonly consumed include chewing tobacco either as *shada* or *zarda*; these are commonly consumed with sliced betel or areca nut and rolled in the *Piper betel* vine or *paan* leaf with slaked lime. Whatever the form of tobacco consumption may be—either chewable or smoke—results of studies have shown their health hazards to be similar (16,17). It is for this reason that all forms of tobacco consumption whether chewed or smoked were categorized together in this analysis.

Two broad categories of independent variables—individual and household—were included in the study. At the individual level, sociodemographic characteristics, such as age; education in terms of years of secular schooling completed which were categorized into none, 1-5 year(s) of schooling, and more than six years of schooling; marital status; and religion, were included. Main occupation was categorized into day labourer or other occupations. Day labourers constituted the poorest of the poor as their work is ill-paid and mostly seasonal. Other occupations included farmers, small traders, self-employed individuals, salaried job holders, housewives, students, and unemployed individuals. At the household level, the sociodemographic characteristics that were studied included educational level and sex of household head, amount of land owned by members of the household, membership of any non-governmental organization, ownership of radio, and location of the household in terms of physical terrain.

Occupation of individuals and ownership of household land were then combined to arrive at a measure of socioeconomic status. Day labourers, irrespective of ownership of land, were characterized as poor, individuals with any other occupation and less than 50 decimals of land were categorized as middle class, and individuals other than day labourers with more than 50 decimals of land were categorized as rich.

### Data analysis

The SPSS software for Windows (version 10.0.1) was used for data management and analysis. Tobacco consumption of the individuals was the dependent dichotomized variable. Both univariate and multivariate techniques of data analysis were performed. In univariate analysis, categorized independent variables were cross-tabulated with dichotomized tobacco consumption to examine the association between the independent variables and tobacco consumption. Logistic regression analysis was carried out with tobacco consumption as a dichotomized dependent variable and all independent variables as categorized variables. A forward stepwise selection method was used for identifying the important variables to arrive at a parsimonious main effect model. Interaction terms were then included one at a time in the main effect model to examine whether the independent variables modified the effects of each other.

## RESULTS

### Background characteristics

Of the 6,618 respondents, 52.1% were males, and 47.9% were females. Approximately 32% of the respondents were aged 15-24 years, while approximately 35% were aged 25-34 years. Over 90% of the study population were Muslims. Almost 62% of the respondents had received no education, and about 34% came from households where the household head was a daily labour, indicating that they were very poor.

### Tobacco consumption

Overall, 43.4% of the study subjects were consumers of tobacco in one form or another. 42.2% of the respondents reported to ‘smoke’ or ‘smoke and chew’, whereas 2.2% only chewed tobacco.

*Bidi* smoking was clearly the predominant form in which tobacco was consumed by both males and females; almost one-third of all males and about 17% of all females consumed it in this form. Cigarettes, the most expensive form of tobacco, followed very closely as the second most common habit among men (around 27%); only 2.7% of all women reported smoking cigarettes. Smokeless forms were more common among females (3.7%) than among males (0.8%).

### Household characteristics and tobacco consumption

Education and occupation of the household head, household land holdings, and ownership of a household radio showed a statistically significant association with tobacco consumption of individuals in univariate analysis. In contrast, sex of the household head and NGO membership of any of the household members showed no significant relationship with tobacco consumption (Table 1).

**Table 1:** Household characteristics and tobacco consumption

Household characteristics	No. of household members	Percentage using tobacco	p value
Sex of household head			
Male	4,458	27.5	0.59
Female	45	31.1	
Education of household head			
None	2,502	32.9	0.00
1-5 year(s)	1,035	24.6	
6+ years	966	16.8	
Occupation of household head			
Day labourer	1,419	32.3	0.00
Other	3,087	25.3	
Household ownership of land			
Landless	1,113	46.7	0.00
Up to 200 decimals	3,948	47.3	
More than 200 decimals	1,557	35.8	
Location of house			
Hilly	1,330	47.2	0.09
High but not hilly	1,105	43.8	
Low	4,134	43.9	
Household ownership of radio			
Yes	1,222	35.9	0.00
No	5,385	46.5	
Any member of household part of NGO group or samity			
Yes	1,895	43.6	0.36
No	4,665	44.9	

Results showed that individuals belonging to households with educated heads consumed less tobacco in proportion to those with illiterate heads. Members of households with a day labourer head were more likely to consume tobacco compared to their counterparts. Consumption of tobacco was lower among members of households owning more than 200 decimals of land than those who were landless.

### Individual characteristics and tobacco consumption

The univariate analysis (Table 2) presents the distribution of tobacco consumers by various individual characteristics. The table shows that sex, age, marital status, years of education, and occupation had a statistically significant relationship with tobacco consumption.

**Table 2:** Individual characteristics and tobacco consumption

Individual characteristics	No. of household members	Percentage consuming tobacco	p value

All	6,618	43.4	-
Sex			
Male	3,448	63.2	0.00
Female	3,170	24.2	
Age (years)				
15-24	2,142	15.3	0.00
25-34	2,288	50.7	
35-44	1,132	70.1	
45+	1,056	63.0	
Religion				
Muslim	5,996	44.9	0.02
Hindu	521	42.0	
Buddhist	101	31.7	
Education				
None	4,096	50.5	0.00
1-5 year(s)	1,346	39.5	
6+ years	1,176	29.3	
Marital status				
Unmarried	1,268	16.9	0.00
Married	5,039	51.4	
Widowed	31	29.0	
Divorced	280	46.8	
Occupation				
Day labourer	1,132	75.5	0.00
Other	5,486	38.1	

Males were almost three times more likely to consume tobacco than females in any form. Consumption of tobacco initially increased with age peaking at around 70% for individuals aged 35-44 years after which it declined.

Completed years of schooling showed a negative relationship with tobacco consumption. Married individuals were the most likely to consume tobacco, followed by divorced and widowed. Individuals having day labour as their occupation were more likely to consume tobacco than others.

Consumption of tobacco was negatively associated with the socioeconomic status of the individuals. Consumption of tobacco decreased with increasing socioeconomic status, and the poor were more than twice as likely to consume tobacco than the rich (Table 3).

**Table 3:** Socioeconomic status of individuals and tobacco consumption

Socioeconomic status	No. of household members	Percentage using tobacco	p value

Poor	1,132	75.5	0.00
Middle-class	2,986	39.9	
Rich	2,500	36.0	

In the logistic regression analysis (Table 4), all the above variables, except religion, continued to have a statistically significant relationship (p<0.001) with tobacco consumption. When the effects of other variables were held constant, males were 9.38 times more likely to consume tobacco than their female counterparts. As in the bivariate situation, individuals aged 35-44 years were most likely to consume tobacco. In a relative sense, individuals with no education were 3.62 times more likely to consume tobacco compared to those who had completed six or more years of schooling. Unmarried individuals were the least likely to smoke. In relative sense, divorced/widowed and married individuals were more than three times as likely to consume tobacco than unmarried individuals. The poor and the middle-class were more likely to consume tobacco than the rich. The poor were almost twice as likely to consume tobacco compared to the rich. The interaction terms involving socioeconomic status and sex, and socioeconomic status and age were not statistically significant at 5%. This implied that the observed relationship between socioeconomic status and tobacco consumption holds true, irrespective of sex and age of the respondents.

**Table 4:** Results of logistic regression analysis of tobacco consumption and individual and household characteristics

Independent variable	Co-efficient	Odds ratio	χ^2^

Constant	-4.82∗∗∗		
Sex			775.86∗∗∗
Male	2.24∗∗∗	9.38	
Female	RC		
Age (years)			25.88∗∗∗
15-24	RC	-	
25-34	1.38∗∗∗∗	3.97	
35-44	1.82∗∗∗	6.14	
45+	1.66∗∗∗	5.24	
Marital status			1.06
Unmarried	RC	-	
Married	1.19∗∗∗	3.29	
Divorced	1.42∗∗	4.14	
Widowed	1.35∗∗∗	3.86	
Education			68.98∗∗∗
No education	1.29∗∗∗	3.62	
1-5 year(s)	0.54∗∗∗	1.71	
6+ years	RC	-	
Religion			2.40
Muslim	0.21	1.23	
Others	RC	-	
Socioeconomic status			23.46∗∗∗
Poor	0.69∗∗∗	1.99	
Middle-class	0.15∗	1.16	
Rich	RC	-	
Wald χ^2^=1780.62; 12 df			

∗∗∗p<0.001;

∗∗p<0.05;

∗p<0.10;

All others non-significant; df=Degrees of freedom; RC=Reference category

## DISCUSSION

Consumption of tobacco in the study area appeared to be more widespread than in other parts of Bangladesh. A nationwide survey reported smoking by 25.2% of the population aged 15 years and above in 1994 (18). The prevalence of smoking among women in the study area was also considerably higher (24.2%) than that noted in other surveys in Bangladesh that have put the figure between 5% and 15% (2,18). Given the prevalent cultural factors that discourage smoking in front of older people in Bangladesh society, it is likely that data collected from household surveys suffer from underreporting of the smoking habit of individuals. Smoking in front of older people is viewed as immodest, and women almost always smoke in private. These same restrictions, however, do not apply to the consumption of chewing tobacco with *paan*.

The inverse relationship between socioeconomic status and tobacco consumption found in this study is consistent with findings from other studies (6,18). In Bangladesh where nearly half of the population live below the poverty line and half of those again below the ‘hard core’ poverty line (19), the high rates of tobacco consumption in these groups have far reaching consequences. Any spending on tobacco from already-constrained household budgets must compete with expenditure on other basic needs of household members (20); concurrently households are placed in a more vulnerable position from the related health problems and their associated economic consequences. Results of a study showed that an estimated 10 million people in Bangladesh currently malnourished could have an adequate diet if money spent on tobacco were spent on food instead (20). Moreover, as the nutrition-mediated effects of smoking, in terms of chronic undernutrition and survival, are likely to be far more important than the direct consequences of smoking on health (21), the Bangladesh population, especially the poorest segment, will be put in a much more vulnerable position. The fact that the poor are also more likely to consume tobacco in its more harmful forms are already more likely to be malnourished and particularly ill equipped to withstand respiratory and other smoking-related diseases and that they are less able to afford healthcare worsens the situation; tobacco-use, thus, may further exacerbate and perpetuate their already vulnerable situation.

The high prevalence of tobacco consumption among women is another major concern facing the nation. Women in Bangladesh are already in poor health and face gender discrimination in terms of seeking healthcare and purchase of medication (22–25). Women are more likely to chew tobacco and to smoke *bidis* with high nicotine content. The health risks of tobacco consumption by women to their children during and after pregnancy are manifold; maternal active smoking has been established to be associated with foetal growth retardation, and women are more susceptible to spontaneous abortion (26–29). Smoking has also been identified as a risk factor for low birthweight in rural Bangladesh (30). Children of women who smoke during and after pregnancy experience abnormalities of infancy and childhood, and more morbidity and mortality (4). Thus, the disadvantageousness of children from socioeconomically-poor households in health and mortality observed in Bangladesh (31,32) are also consistent with the socioeconomic patterns of tobacco consumption.

Considering the impact of involuntary smoking, the problem may even be greater for it is likely that neither smokers nor non-smokers are aware of its consequences. It is believed that exposure to environmental tobacco smoke (ETS), the smoke emitted from a lit cigarette and tobacco smoke exhaled by the smoker, is associated with a higher risk of lung and respiratory diseases (4) and with several other important health ailments in children, including sudden infant death syndrome. Unborn children are also put at risk when pregnant women are exposed to ETS, increasing the chance of low birthweight, intrauterine growth retardation, and preterm delivery (3). Although smoking in public places, such as buses and trains, are restricted, adherence to this restriction by the public is minimal. Moreover, it is not known to what extent people are aware about ETS and what measures they take to minimize the effects of ETS in the workplace and at home.

Within this backdrop, the challenge is how to reduce the prevalence of tobacco consumption among the population in general and among the vulnerable groups in particular. It may be useful to examine factors that may support and discourage smoking in the context of Bangladesh. Consumption of tobacco—both smoking and chewing—has been a long-standing tradition in Bangladeshi households (21). Tobacco and/or *paan* are quite often the only items, which are offered to visitors, or when meeting with a friend. Tobacco in chewable form is a very common household consumption item and is always offered to a visitor—male or female. A Bangladeshi household without tobacco in some form or other is hard to imagine. The only factor that discourages tobacco consumption is the culturally inappropriateness of younger people smoking in front of elders; even adult offspring do not smoke in front of their parents. However, smoking is hardly perceived as a health hazard.

There have been some statutory measures enacted in relation to advertisements of smoking products to discourage smoking. Each advertisement of cigarettes or *bidis* now includes a message stating that smoking is harmful to health. Health warnings are also mandatory on packaging of cigarettes and *bidis*. However, in a country where half of the population is illiterate, one cannot be sure about the effectiveness of these printed messages in discouraging smoking. Moreover, a good proportion of cigarettes and *bidis* are sold singly and not by the packet; only those who purchase a full packet and can read may benefit from these warnings; customers who buy singly are likely to miss the warning on the packet completely. In addition, the legislative provisions discourage smoking and not tobacco consumption per se, i.e. consumption of tobacco in chewable forms is not discouraged by the existing measures. Another aspect that may encourage production and consumption of tobacco is the aggressive role of tobacco companies, particularly in commissioning cultivation of tobacco. The industries pay farmers in advance to cultivate tobacco and ensure their marketing. Those engaged in cultivation of tobacco may also be a good consumer of their own product. Environmental damage is caused during the drying of the tobacco leaves in locally-designed dryers; the firewood used is collected from nearby forests leading to deforestation and soil erosion, while the burning itself produces smoke.

Conversely, cultivation of tobacco provides an important and steady cash-earning opportunity for small farmers. Production of tobacco creates employment in rural areas where employment is scarce. In the absence of a comparative analysis of the social and economic costs of prohibiting tobacco cultivation, it is hard to make firm comments on this.

Despite the seriousness of public-health consequences of tobacco consumption in general and for the poor in particular and the complexity of the political economy of the tobacco industry, the existing knowledge in this area is limited in Bangladesh. Further research on this topic can help narrow down the knowledge gap and, therefore, initiate policies to combat the detrimental effects of tobacco consumption.

One approach to combat tobacco consumption could be massive anti-tobacco campaigns aimed at influencing policy and individual behaviour. Such efforts should make the use of the lessons learned from other nations of the world. It is only through aggressive anti-tobacco programmes in various fronts that there may be a chance of salvaging the vulnerable groups from the menace of tobacco consumption in Bangladesh.
